# Acceptability of mandatory tuberculosis notification among private practitioners in Yogyakarta, Indonesia

**DOI:** 10.1186/s13104-019-4581-9

**Published:** 2019-08-27

**Authors:** Ari Kurniawati, Retna S. Padmawati, Yodi Mahendradhata

**Affiliations:** 1 Postgraduate Programme in Public Health, Faculty of Medicine, Public Health and Nursing, Universitas Gadjah Mada, Yogyakarta, Indonesia; 2 Department of Health Behaviour, Environment, and Social Medicine, Faculty of Medicine, Public Health and Nursing, Universitas Gadjah Mada, Yogyakarta, Indonesia; 3 Department of Health Policy and Management, Faculty of Medicine, Public Health and Nursing, Universitas Gadjah Mada, Yogyakarta, Indonesia

**Keywords:** Tuberculosis, Mandatory notification, Acceptability, Private practitioners, Indonesia

## Abstract

**Objective:**

Indonesia ranks second globally in the number of cases not reported to the National Tuberculosis Control Program, accounting for 11% of the total cases lost worldwide. In 2016, the Ministry of Health has issued Regulation Number 67 on tuberculosis control, which requires mandatory tuberculosis notification. We aimed to assess the prospective acceptability of mandatory tuberculosis notification among solo private practitioners and private primary care clinics in Yogyakarta.

**Results:**

Our study highlighted critical issues which need to be addressed in ensuring acceptability of mandatory tuberculosis case notification. We found that that private practitioners do not notify tuberculosis cases due to a lack of policy knowledge. Mandatory tuberculosis notification and its potential penalties were also felt as burdensome by private practitioners. There were ethical concerns among the private practitioners in our study about patient’s privacy and patients potentially lost to other healthcare facility. Private practitioners emphasized the need for intervention coherence and cooperation. We also observed pattern variations of these constructs across characteristics of private practitioners.

## Introduction

Tuberculosis (TB) is globally one of the top ten causes of death and the leading cause from a single infectious agent [1]. In 2017, 6.4 million new cases of TB worldwide were formally reported to national TB control programs and then notified to the World Health Organization (WHO). As 10.0 million new cases have been estimated to occur in 2017, these reported 6.4 million cases reported only represented 64% of the estimated total new cases [1]. Thus, approximately 3.6 million cases are actually missed globally [1]. A combination of under-diagnosis and under-reporting of detected cases contribute to the gaps between the estimated number of new cases and the number notified [1].

Indonesia has the third highest TB burden in the world with 842,000 estimated cases occurring in 2017 [1]. Indonesia ranks also second globally in the number of cases not reported to the National TB Program, accounting for 11% of the total cases lost worldwide [1]. The Joint External Monitoring Mission 2017 reported that while around 7500 of the notified cases die, it is estimated that more than 100,000 deaths annually occur as unreported cases [2]. The majority of these missing cases are estimated to be in the private sector and unreported despite patients being able to access initial diagnosis and treatment [2, 3].

Evaluation of 98,000 Indonesian Medical Association members indicated that 7000 had participated in International Standard of Tuberculosis Care (ISTC) education programs by 2010, but only 1342 consistently reported TB case findings [4]. Mahendradhata et al. [5] found substandard management of TB cases among private practitioners in Indonesia. Stronger regulation is needed to ensure standard TB diagnosis, treatment and notification from the private sector [6]. The Ministry of Health has subsequently issued Regulation No. 67 of 2016 on tuberculosis control [7], which stipulated mandatory TB notification for private clinics and solo practices, with consequences of penalties for incompliance. Successful implementation of such policy arguably depends on its acceptability. The objective of this study is to explore the prospective acceptability of the TB mandatory notification policy, among solo private practitioners (PPs) and private primary care clinics in Yogyakarta municipality, Indonesia.

## Main text

### Methods

We conducted an explorative qualitative study to describe prospective acceptability of the mandatory notification policy of TB among solo PPs and private primary care clinics, in accordance with the Theoretical Framework of Acceptability (TFA) [8]. The qualitative approach has been chosen as it is most appropriate to answer questions about experience, meaning and perspective, most often from the standpoint of the participant [9]. These data are usually not amenable to counting or measuring [9].

The study is conducted in the setting of the Yogyakarta municipality, which is the capital of Yogyakarta Special Province, situated in southern part of the Java Island. The municipality covers an area of 32.5 km^2^ with a population of 409,487 [10]. Table 1 presents a summary of public and private health care facilities in Yogyakarta municipality [10]. There are currently 176 registered solo PPs in Yogyakarta; 55 of them are specialists while the rest are general practitioners (GPs). Additionally in Yogyakarta there are 17 private primary clinics that collaborate with the national health insurance agency. The National health insurance agency, which was established in 2014, has established TB case management guideline [11].

**Table 1:** Public and private healthcare facilities in Yogyakarta Municipality [10]

Public healthcare facilities	Number	Private healthcare facilities	Number
Health Center without inpatient	15	General Hospital	7
Health Center with inpatient	3	Specialized Hospital	4
Satellite Health Center	8	Mother and Child Hospital	5
Civil General Hospital	2	Health Clinic	52
Military General Hospital	1	Beauty Clinic	21
Military Clinic	2	Solo Private Medical practice	176
Police Clinic	2	Solo Private Dental practice	73
		Midwifery practice	13
		Private Clinical Laboratory	15
		Pharmacy	129
		Traditional medicine practice	113

In line with the mandatory TB notification policy stipulated by the Minister of Health Regulation No. 67 of 2016, the Yogyakarta municipal government launched the Yogyakarta Regional Action Plan for TB Control 2017-2021 as stipulated in the Mayor Regulation No. 102 of 2017 [12]. This regulation calls for the increased involvement of private practitioners in TB case mandatory notification and intensification of TB case finding through training for private primary care centers and solo PPs.

We employed purposive sampling, rather than probabilistic sampling, for identifying and selecting individuals that are especially knowledgeable about or experienced with phenomenon of interest, i.e. mandatory TB notification [13]. We specifically used the maximum variation sampling strategy by identifying key dimensions of variations (e.g. characteristics of medical practice, medical qualification) and then finding cases that vary from each other (e.g. solo practitioners vs private clinic practitioners; general practitioners vs specialists) as much as possible [14]. This allowed us to document unique or diverse variations that have emerged in adapting to different conditions [13].

We initially recruited PPs based on the maximum variation sample constructed from the PPs register of the municipal health office, and cross-checked with the list from the medical association. We contacted the selected PPs and provided information on the study background, procedure and method before obtaining consent. Based on the information from PPs who were interviewed initially, we identify other potential informants to be recruited. As qualitative research approach place primary emphasis on information saturation, we continued to sample until no new substantive information is acquired, rather than following sample size estimation [13].

In-depth interviews were carried out from March to April 2018 by the first author (AK), who has been formally trained in qualitative research method, under supervision of a qualitative research expert. The in-depth interviews were informed by an interview guide which were developed in consultation with qualitative research expert and tested beforehand with solo PPs practicing outside the Yogyakarta municipality. Questions in the interview guide focused on how PPs feel about participating in the TB mandatory notification policy; how much effort (time, effort, cost, thought) is felt to be needed to participate in TB mandatory notification policy; to what extent the TB mandatory notification policy has good conformity with the value system adopted by PPs; and how is PPs’ understanding about the TB mandatory notification policy in advance and how the mandatory notification of TB cases works. The interview took approximately 1 h to complete. Each interview was audio recorded. Verbatim transcripts were developed based on the recoding and interview notes. We conducted thematic analysis guided by the relevant constructs of TFA [8]: affective attitudes, burdens, ethics, and coherence of interventions. To ensure trustworthiness, we performed triangulation, debriefing and member checking [15].

### Results

We interviewed a total of 28 informants (Table 2) consisted of 9 solo PPs; 8 private clinic physicians and 11 TB program stakeholders (e.g. municipal health offices, primary health care centers, donor project technical officer).

**Table 2:** Characteristics of study informants

	Private practitioners		Stakeholders				
	Solo PP (n = 9)	Private Clinic Physician (n = 8)	City Health Office (n = 2)	Provincial Health Office (n = 2)	Donor project Technical Officer (n = 2)	Primary Health Centers (n = 4)	Medical Association (n = 1)
Gender							
Male	3	2	0	1	1	1	0
Female	6	6	2	1	1	3	1
Age							
25–34	1	3	0	0	0	0	0
35–44	3	2	0	0	0	2	0
45–54	2	1	2	2	0	2	
55–64	2	1	0	0	1	0	1
< 65	1	0	0	0	1	0	0
Academic background							
Diploma	0	0	0	0	0	1	0
Undergraduate	0	0	1	0	0	1	0
Master degree	0	0	0	2	2^a^	0	0
Medical Doctor	5	6	1	0	2^a^	2	0
Specialist	4	2	0	0	0	0	1
Working experience (years)							
> 5	1	3	0	0	0	1	0
5–10	2	3	0	1	0	3	1
> 10	6	2	2	1	2	0	0

^a^Medical doctor with master degree

Our interview data describe the prospective acceptability of mandatory TB case notification among solo PPs and private primary clinics, based on TFA constructs: affective attitude, burden, ethicality, and intervention coherence of mandatory TB notification. For each of these constructs, our results revealed general patterns as well as particular patterns across different characteristics of private practitioners.

#### Affective attitudes

This study found that PPs in Yogyakarta generally felt involved in the management of TB cases, but most did not report due to ignorance about the importance or lack of education concerning TB mandatory notification policy. This was also the case even for PPs who during the day served as civil servants and are in regular contact with the public primary health centers. The following excerpts from the interviews reflect this lack of policy knowledge.

*“It’s true that TB is also a government program, territorial actually…… But yes in this city I have never seen or heard or disseminated about this [mandatory notification policy], there is no term on how I should report if there is a case of TB”*

(GP, Solo private practice)

*“In my [practice] at home, I reported nothing….at the hospital there is already a system, there is a form for this.”*

(Specialist, Solo private practice)

*“There was a TB training in 2013…in the end it was agreed that TB suspect should be sent to health centers to be managed. Unfortunately there has been no follow up [of this agreement]”*

(GP, Solo private practice)

*“Back then we knew that patients with coughs more than 2* *weeks meet criteria for TB tracing, but…I don’t know the reporting procedure. I have never followed any TB training or dissemination”*

(GP, Private clinic)

#### Burden

This study found that mandatory notification of TB is generally quite burdensome for solo PPs both in terms of human resources and sanctions. Some argue it would not be a heavy burden, if there is a notification system and mechanisms that are easy and practical. The following excerpts from the interviews reflect this sense of burden and provide possible solutions.

*“It’s actually about human resources. When private practitioners cannot do it, it [reporting] can be delegated to medical record staff….If the practice is part of a clinic may be possible, but if for solo practice may be burdensome.”*

(GP, Private clinic)

*“It is a big burden if the sanction is like that. ….. Maybe this rule must have requirements, explanations or disseminated first”*

(GP, Private clinic)

*“Reporting should be easy as there is not many cases. As long as there is a form and we do not have to make it ourselves”*

(GP, Private clinic)

*“ …the forms should be provided … softcopy or hardcopy…once a month…sent by email. Or perhaps with [mobile phone] applications would be more practical…”*

(GP, Solo private practice)

#### Ethicality

The problem of ethicality encountered by some solo private practitioners involved physician–patient confidential relationships or relationships with other parties such as Primary Health Centers. There was also concerns over the possible risk of sharing of TB patient data (violation of privacy) for reporting purposes, or the risk of patients lost to other care providers. The following excerpts reflect a few ethical considerations which arose from the TB notification policy.

*“Yes, so later if we report and then the patient knows….he is angry to us, it is also troublesome*

(GP, Solo private practice)

*“If the problem is about ‘competing’ for patients, well what’s important is communication… ”*

(GP, Solo private practice)

*“The concern that [their] patients are being taken away is understandable, certainly there is business interest in private [sector]”*

(Officer, Municipal health office)

*“Actually they will not just lose one patient [to health center], but many. One family may be ‘lost’, even the neighbors, when they hear from mouth to mouth that [private practitioner] cannot treat TB….”*

(Provincial Technical Officer, International donor project)

#### Intervention coherence

The study also found that solo private practitioners and private primary clinics, as well as early stakeholders generally understood that mandatory TB notification is an important public health policy that contributes to improving case finding and is aimed to reduce drop-out rates for TB patients. They are ready to cooperate and contribute when there is a clear system and practical mechanism. The following excerpts reflect this need for intervention coherence and cooperation with the new TB notification policy.

*“I do not think there’s any additional obligation for this notification. Because after all, if not reported eventually can’t be traced”*

(GP of private primary clinic)

*“There is no policy, for example in the city, that each of the Primary Health Center has to coordinate with the private sector in the area“*

(Policy maker of Yogyakarta Health Office)

*“For officially coordinating solo private practices and private clinics, frankly we do not dare yet because there is no mechanism and instruction yet from the [municipal] health office, we don’t want to bypass them, we’re ready if [the mechanism and instruction are] clear”*

(Head, Health Center)

We observed some pattern variations of these constructs across characteristics of PPs (Table 3). Among GPs in solo private practices, the seniors notably expressed more burden and ethicality of mandatory TB case notification. Those who also served as civil servant during the day notably expressed less burden and ethicality of mandatory TB case notification. Among the specialists, burden and ethicality of mandatory TB case notification is more expressed by the pulmonologists.

**Table 3:** Summary of the prospective acceptability of mandatory TB notification among private practitioners in Yogyakarta, Indonesia

Category	Sub category	Affective attitude of mandatory TB notification	Burden of mandatory TB notification	Ethicality of mandatory TB notification	Intervention coherence of mandatory TB notification
General practitioner (solo private practitioner)	Senior	+−	+++	+−	+++
	Junior	− − −	− − −	− − −	+++
	High patient caseload	+−	+−	− − −	+++
	Low patient caseload	− − −	+−	− − −	+++
	Government employees	− − −	+−	+−	+++
	Non-government employees	+−	+−	+++	+++
General practitioner (private primary clinic)	Senior	+−	+−	− − −	+++
	Junior	+−	+−	− − −	+++
	High patient caseload	+−	+−	− − −	+++
	Low patient caseload	− − −	+−	− − −	+++
Specialist (solo private practitioner)	Pulmonologist	− − −	+++	+++	+++
	Pediatrician	− − −	− − −	− − −	+++
	Internist	+−	− − −	− − −	+++

### Discussion

Our study highlighted critical issues which need to be addressed in ensuring acceptability of mandatory TB case notification. We found that that PPs may not notify TB cases due to a lack of policy knowledge. A study in India also highlighted that introduction of PPs to the notification program and their understanding about the importance and support for the notification program strongly determined their long-term involvement [16].

Mandatory TB notification and its potential penalties were also felt as burdensome by PPs in our study. In general, participation in any intervention involves time and cost, and often much cognitive effort, to foster individual confidence in engaging in a new intervention [8]. Thomas et al. [16] suggest that to facilitate effective TB notifications among solo PPs, user-friendly intervention strategies with different options can be used, especially smartphone media, such as direct telephone calls, SMS, or applications. Another study in India demonstrated that online case reporting platform improved accessibility to reporting for private providers, however remains time-consuming and require the support of an intermediary NGO to deal with the amount of required data [17].

There were ethical concerns among PPs in our study about patient’s privacy and patients potentially lost to other healthcare facility. In line with our finding, Thomas et al. [16] also found that some PPs feared that the notifications would lead to privacy concerns or interfere with patient confidentiality. Chowdhury et al. further argued that TB mandatory notification breach the patients’ right to confidentiality and that is particularly a matter of concern in a society where TB is widely stigmatized [18].

Additionally our findings emphasized the need for better intervention coherence. In line with Phalkey et al.’s [19] recommendations, the involvement of PPs requires periodic training, supportive supervision, and regular feedback to improve PPs’ compliance with the mandatory TB notification policy. Uplekar [20] described five practical components required to facilitate a fully operational mandatory TB notification system to work optimally: policies and regulations, systems and reporting mechanisms, service provider orientation, feedback, and monitoring and evaluation. A study in South India also highlighted the need for a complete and coherent approach for enhancing notification, including improving healthcare providers’ awareness regarding TB notifications requirement, establishing a single notification portal, digitally linking medical records and appointing one person to be responsible for notification [21]. There were notably some pattern variations of our findings across different characteristics of PPs. This underlines the need to customize the engagement approach to the characteristics of targeted PPs.

Therefore, our study highlighted important steps to be taken by key local actors, such as TB control program officers, who should pay particular attention to affective attitudes, burden, ethicality, and intervention coherence in order to ensure acceptability of mandatory TB notification policy. Enhancing affective attitudes and communications may alleviate the burdens and overcome ethical concerns related to mandatory TB notification. Coherence of interventions since the beginning of implementation is an especially important capital for the acceptability of mandatory TB notification at the next stages of implementation. There is a critical need to actively engage solo private practitioners in the mandatory TB notification policy. The Municipal Health Office and heads of the Primary Health Centers thus must strengthen their internal capacities to more systematically engage private practitioners and collaborate with key stakeholders.

Reflections from applying the Theoretical Framework of Acceptability [8] in our study may also contribute to further refine the framework. We found some TFA constructs to be overlapping especially between affective attitudes and intervention coherence, so some adjustments are recommended in the application of these constructs. We also recommend to further develop this theory by adding other external or environmental factors which influence the acceptability of new interventions.

## Limitations

Our study have limitations which need to be considered to avoid overgeneralization. Firstly, we only conducted the study in Yogyakarta municipality, thus the findings should be interpreted prudently when considered in different settings. Secondly, not all of PPs we approached agreed to participate and there were limited participation among specialists. Thirdly, not all Heads of the Primary Health Centers could be interviewed. Thus, in order to minimize potential information gaps, we conducted additional interviews with key officers at the provincial health office who have played various key roles in the Public Private Mix TB program for more than 10 years.

## Data Availability

The datasets used and/or analysed during the current study are available from the corresponding author on reasonable request.

## Abbreviations

GP: general practitioners
ISTC: International Standards for Tuberculosis Care
PP: private practitioners
TB: tuberculosis
TFA: Theoretical Framework of Acceptability
